# Association of α/β-Hydrolase D16B with Bovine Conception Rate and Sperm Plasma Membrane Lipid Composition

**DOI:** 10.3390/ijms21020627

**Published:** 2020-01-17

**Authors:** Shuwen Shan, Fangzheng Xu, Martina Bleyer, Svenja Becker, Torben Melbaum, Wilhelm Wemheuer, Marc Hirschfeld, Christin Wacker, Shuhong Zhao, Ekkehard Schütz, Bertram Brenig

**Affiliations:** 1 Institute of Veterinary Medicine, University of Goettingen, 37077 Goettingen, Germany; 2 Pathology Unit, German Primate Center, Leibniz-Institute for Primate Research Goettingen, 37077 Goettingen, Germany; 3 Department of Obstetrics and Gynecology, University Medical Center Freiburg, 79106 Freiburg, Germany; 4 Key Lab of Animal Genetics, Breeding and Reproduction, College of Animal Science and Technology, Huazhong Agricultural University, Wuhan 430070, China

**Keywords:** Holstein cattle, male infertility, ABHD16B

## Abstract

We have identified a Holstein sire named Tarantino who had been approved for artificial insemination that is based on normal semen characteristics (i.e., morphology, thermoresistance, motility, sperm concentration), but had no progeny after 412 first inseminations, resulting in a non-return rate (NR*_dev_*) of −29. Using whole genome association analysis and next generation sequencing, an associated nonsense variant in the α/β-hydrolase domain-containing 16B gene (*ABHD16B*) on bovine chromosome 13 was identified. The frequency of the mutant allele in the German Holstein population was determined to be 0.0018 in 222,645 investigated cattle specimens. The mutant allele was traced back to Whirlhill Kingpin (bornFeb. 13th, 1959) as potential founder. The expression of *ABHD16B* was detected by Western blotting and immunohistochemistry in testis and epididymis of control bulls. A lipidome comparison of the plasma membrane of fresh semen from carriers and controls showed significant differences in the concentration of phosphatidylcholine (PC), diacylglycerol (DAG), ceramide (Cer), sphingomyelin (SM), and phosphatidylcholine (-ether) (PC O-), indicating that ABHD16B plays a role in lipid biosynthesis. The altered lipid contents may explain the reduced fertilization ability of mutated sperms.

## 1. Introduction

Fertility is an important economical productivity factor in animal breeding [1,2,3,4]. Indicators to assess male fertility can either be indirect (e.g., productivity of progeny, sire conception rate, non-return rate) or direct (e.g., semen characteristics, testis size) [5,6,7,8,9,10]. The latter parameters have the advantage that they can be easily measured and they provide an immediate answer; however, the heritabilities of scrotal circumference and semen traits vary extremely, ranging from 0.0 (i.e., abnormal heads, bent tails, distal cytoplasmic droplets) to 0.57 (i.e., scrotal circumference) and, therefore, their use in selection is not always straightforward [11]. Alternative approaches were used to determine the differences between fertile and infertile bulls while using molecular tools. Transcriptome analyses for instance have shown that spermatozoa of high-fertility bulls show a higher concentration of specific transcripts for membrane and extracellular space protein locations [12,13]. In another study residual RNA content in spermatozoa of bulls with extreme non-return rates was analysed [14]. Low-fertile bulls showed a significantly increased amount of ribosomal and mitochondrial sequences, whereas high-fertile bulls exhibited transcripts of genes that are involved, for example, in metabolism, signal transduction, translation, and protein degradation [14]. From transcriptome and proteome studies, mainly in man, mouse, and rat, it is evident that differences between RNA and protein content, DNA methylation, posttranslational modifications between fertile and infertile individuals exist [15,16,17,18]. The use of these types of biomarkers in reproductive medicine is believed to bridge the gap between conventional semen analysis with limited clinical utility and biochemical pathways that regulate male fertility [19].

However, the assessment of mutational effects in candidate genes is normally challenging, especially when there are only subtle deviations in expression levels, due to the complex interactions of geno- and phenotypes in fertility traits [20,21]. With the advancement of high-throughput screening tools (DNA chip, next generation sequencing) and the availability of large datasets on fertility parameters of bulls, especially in Holstein cattle male fertility, can be practically implemented into genomic selection [22]. Genome-wide association studies have been conducted in Holstein bulls, identifying several fertility associated genomic regions [23]. A recent genome-wide association study has detected at least eight genomic regions, i.e., on bovine chromosome 5 (BTA5), BTA9, BTA13, BTA21, and BTA25, in Holstein cattle associated with bull fertility while using Sire Conception Rate (SCR) as a parameter [24]. In a large multi-species comparative study 33 promising candidate genes have been identified for male fertility/infertility [25]. Recently, a whole exome sequencing of 24 high and low fertile bulls identified 484 SNPs that were significantly associated with fertility [26]. The second most significantly associated SNP in this study was located on BTA13 at position 53,691,419 within the *SIRPA* gene. Although these data point at a number of potential molecular targets only three causative mutations, resulting in male sub- or infertility in cattle have been determined in the *FSHB*, *TMEM95*, and *ARMC3* gene hitherto [27,28,29,30].

Here, we report about the identification of nonsense variant in the bovine α/β-hydrolase D16B gene (*ABHD16B*) on BTA13 significantly associated with male subfertility in Holstein cattle. So far, nothing was known regarding the physiological or biochemical function of ABHD16B [31,32]. Our data provide evidence that ABHD16B is involved in lipid biosynthesis in testis and is crucial for fertilization.

## 2. Results

### 2.1. Conception Ability of Sires Is Highly Associated with a Chromosomal Region on Bovine Chromosome 13

A Genome Wide Association Analysis (GWAS) was performed while using a cohort of 289 Holstein sires to determine chromosomal regions harboring associated causative genes for conception ability (NR*_dev_*). The cohort consisted of 10 sires with a NR*_dev_* ≤ −2 (= cases) (Table 1) and 279 randomly chosen sires of the active breeding population (= controls).

**Figure 1:**
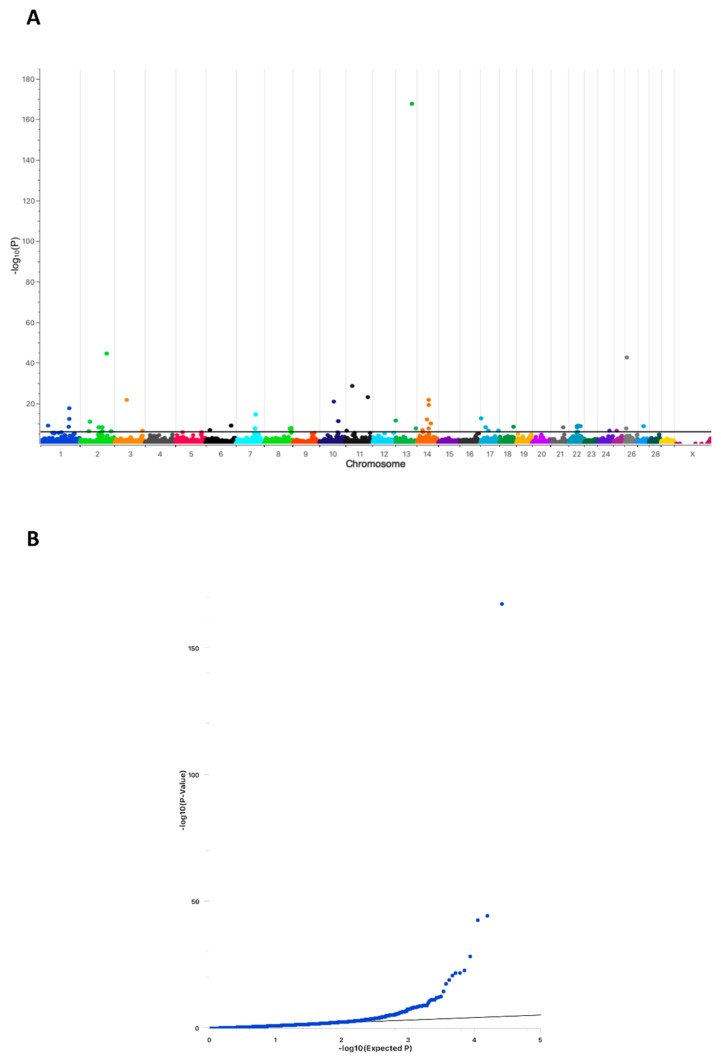
Manhattan plot of the Genome Wide Association Analysis (GWAS) (*n* = 289; 279 controls, 10 cases). (**A**) The plot shows the -log_10_-transformed *p*-values for all SNPs. The black horizontal line represents the genome-wide significance threshold of −log_10_*P* = 5.9. (**B**) Quantile-quantile (QQ) plot of the GWAS.

Individual NR*_dev_* values of the control sires were not available; however, they were assumed to be normal, as all of these sires were used in the current breeding population. As shown in Figure 1A, one genome-wide highly significant associated position on BTA13 (ARS-BFGL-NGS-107931; position 63,500,701) was detected (−log_10_*P*-value = 167.56). Seventeen additional regions above a Bonferroni threshold of −log_10_*P* = 5.9 (*p* < 0.01) with much lower significance were present on BTA1, 2, 3, 6, 7, 8, 10, 11, 14, 17, 18, 21, 22, 24, 25, 26, and 27. The QQ-plot clearly indicated a compelling evidence for an excess of association with no population substructure (Figure 1B). Whole-genome sequencing was performed while using Tarantino and his parents to determine which of the associated chromosomal regions harbored protein-altering variants that were causative for Tarantino’s infertility.

### 2.2. Whole-Genome Sequencing Reveals Two Potential Protein-Altering Variants Upstream the Associated Position on BTA13

Raw next generation sequencing data were quality filtered. Within the filtered 78,472 SNPs, only 20 resulted in a predicted loss of function, including 10 nonsense variants, five splice-donor variants, three splice acceptor-variants, and two initiator-codon variants. Two SNPs were located near the associated position on BTA13, i.e., a nonsense variant at position 54,429,815 within the single exonic α/β-hydrolase D16B (*ABHD16B*) gene (AC_000170.1: g.54429815G>A, rs468948776) and a splice-acceptor variant at position 53,003,648 within the transmembrane channel-like protein 2 (*TMC2*) gene (AC_000170.1: g.53003648C>T, rs465702794). *TMC2* has been shown to be expressed in the inner ear and it is necessary for the mechanotransduction in cochlear hair cells [33,34]. *TMC2* was excluded as potential candidate due to this very specific function. On the other hand, *ABHD16B* has been shown in humans to be mainly expressed in testis, which suggested a potential role in Tarantino´s infertility [35]. In addition, aberrant methylation patterns of *ABHD16B* have been shown to be associated with infertility in men [36].

### 2.3. Verification and Validation of the Nonsense Variant g.54429815G>A (ABHD16B) in the Holstein Population

An initial set of 2072 randomly selected Holstein DNA samples were genotyped to verify and validate the presence of the detected variant in *ABHD16B*. In this set, 2052 wild type (G_G), 20 heterozygous (G_A), and no homozygous (A_A) carrier were detected (HWE χ^2^= 0.05). The results proved that the variant was present in the population at a very low frequency. Therefore, are larger cohort of 222,645 HF cattle (208,165 female, 14,480 male) was genotyped while using the bovinSNP50 BeadChip. In this cohort, 810 heterozygous (781 female, 19 male, 10 unknown sex) and no homozygous animals were identified, resulting in a frequency of the variant allele of 0.0018. According to Hardy—Weinberg equilibrium it was not unexpected that no homozygous individuals were detected (HWE χ^2^= 0.73). The low allele frequency further supported the data that the nonsense variant in *ABHD16B* was most likely the causative variant for Tarantino´s infertility, because sires will be rapidly removed from the breeding population once a sub- or infertility would have been evident during routine fertility testing. Such a selection will efficiently reduce the transmission and spreading of the causative variant. The limited number of heterozygous individuals in the randomly chosen large Holstein cohort prompted us to determine the number of heterozygous sires in the available DNA samples of Tarantino´s close male relatives in correlation with their conception ability (NR*_dev_*). A total of 34 DNA samples were available and genotyped, resulting in 16 wild type and 18 heterozygous sires (HWE χ^2^= 4.4). Within the heterozygous sires, 15 had negative NR*_dev_* values (−9 to < 0) and only three sires showed positive NR*_dev_* values (0 to 2).

### 2.4. Expression and Tissue Distribution of ABHD16B

**Figure 2:**
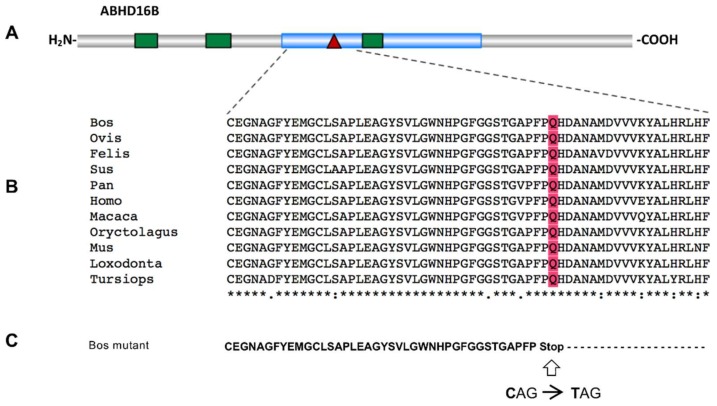
(**A**) Schematic representation of the ABHD16B protein structure indicates the position of the α/β-hydrolase fold domain (blue) and the transmembrane helices (green), predicted by NCBI Conserved Domains Database and TMbase, respectively. The amino acid position (218) of the nonsense variant leading in a premature stop is marked by a red triangle. (**B**) The comparative alignment of amino acid sequences of 11 mammals while using Clustal W (178-amino acid position 178–237) is shown. The amino acid position at the truncation site is indicated in red. NCBI protein sequence accession numbers are as follows: Bos (*Bos Taurus*) NP_001033630.1, Ovis (*Ovis aries*) XP_014955258.1, Felis (*Felis catus*) XP_003983341.3, Sus (*Sus scrofa*) XP_020933693.1, Pan (*Pan troglodytes*) XP_003317106.1, Homo (*Homo sapiens*) NP_542189.1, Macaca (*Macaca mulatta*) NP_001180656.1, Oryctolagus (*Oryctolagus cuniculus*) XP_008250767.2, Mus (*Mus musculus*) NP_899004.1, Loxodonta (*Loxodonta Africana*) XP_003421827.1, Tursiops (*Tursiops truncates*) XP_019806804.1. (**C**) The amino acid sequence of bovine ABHD16B truncated protein with the stop-gain variant.

*ABHD16B* codes for a protein of 470 amino acids with a predicted α/β-hydrolase fold domain. The nonsense variant g.54429815G>A causes a premature stop at amino acid position 218 (glutamine residue), resulting in a truncation of 253 C-terminal amino acids and 53.8% of the protein (Figure 2). In silico protein sequence comparison of 11 mammals revealed that the glutamine residue (Q) is highly conserved. Due to the truncation 67.4% of the α/β-hydrolase fold domain is missing. Regarding the evolutionary appearance, it is interesting to note that *ABHD16B* first evolved in reptiles performing internal fertilization. Species with external fertilization, e.g., fish and frogs, do not harbor an *ABHD16B* gene.

### 2.5. ABHD16B Is Expressed in Testis but not in Spermatozoa

**Figure 3:**
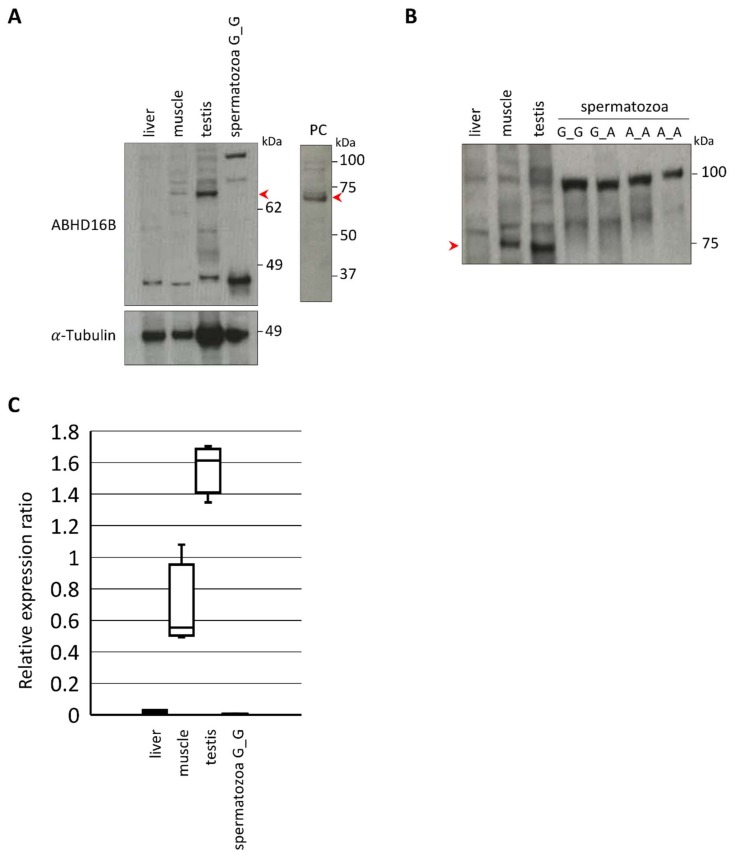
Western blot analysis of ABHD16B protein expression in tissues and spermatozoa. (**A**) ABHD16B protein band (red arrow) detected in wild type testis (G_G) but not in spermatozoa. Using liver as negative control, muscle and human ABHD16B over-expression lysate as positive controls (PC). α-Tubulin used as the loading control. (**B**) ABHD16B (approx. 70 kDa) is absent in spermatozoa of three genotypes (wild type (G_G), heterozygous (G_A) and homozygous carrier (A_A)). Spermatozoa of homozygous carriers were from Tarantino and C_a_. (**C**) Box and Whisker plot of relative ABHD16B expression. Areas under curve were determined using ImageJ 1.52k software and relative expression ratios of ABHD16B (%) in liver, muscle, testis, and spermatozoa (G_G) were calculated while using α-Tubulin expression as internal standard. Horizontal lines within boxes indicate median values and whiskers show upper and lower extremes.

Western blotting was used to detect ABHD16B in testis (wild type) and spermatozoa (wild type, heterozygous, and homozygous variant) extracts. Testes of heterozygous and/or homozygous carriers were unavailable due to the low genotype frequencies. However, a limited amount of deep-frozen semen samples of Tarantino and a further not directly related homozygous carrier (C_a_) provided from the safety inventory of an AI station were included in the analysis. An ABHD16B specific band was detected in testis of wild type bulls at the expected size of approx. 70 kDa, as shown in Figure 3. Neither in wild type nor in heterozygous or homozygous variant spermatozoa extracts ABHD16B was detected (Figure 3B). 

### 2.6. Immunohistochemical Analysis Revealed ABHD16B Expression in Testis and Epididymis

**Figure 4:**
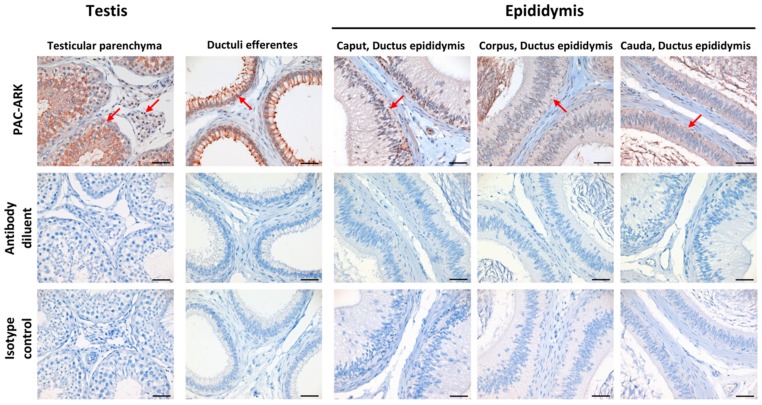
ABHD16B protein detection and localization in testis and epididymis of wild type bull by immunohistochemistry. PAC-ARK antibody was used as primary antibody. Positive staining is indicated with red arrows. When the primary antibody (PAC-ARK) was replaced with antibody diluent and isotype rabbit IgG at the same working dilution, no staining was observed in any of these tissues. Scale bars = 50 µm.

Sections of testicular and epididymal tissue samples that were collected at an abattoir were prepared for IHC. The *ABHD16B* genotype of the samples was tested prior to IHC and shown to originate from wild type sires. While using the PAC-ARK antibody, ABHD16B expression was detectable in testicular parenchyma, ductuli efferentes, as well as epididymal tail, body, and head, as shown in Figure 4. Specifically, there is ABHD16B expression in the nucleoplasm of Leydig cells, in the seminiferous tubules and, with variable intensity, in the epithelium of the ductus epididymis. These findings suggest that ABHD16B probably plays a role in spermatogenesis and sperm maturation.

### 2.7. ABHD16B Is Involved in Lipid Metabolism and Influences Sperm Plasma Membrane Lipid Composition

**Figure 5:**
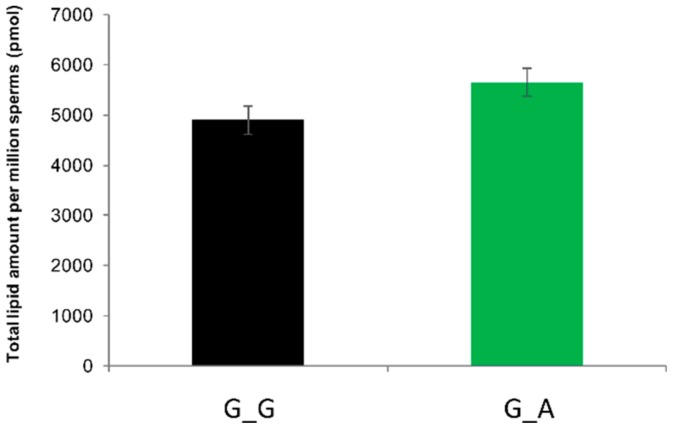
Total lipid amount of 10^6^ sperms of each genotype revealed no significant difference after normalization. G_G: wild type; G_A: heterozygous carrier.

We hypothesized that ABHD16B could be involved in plasma membrane lipid biosynthesis, as many members of the α/β-hydrolase superfamily of hydrolytic enzymes are involved in lipid metabolism. Sperm lipidomics of heterozygous and wild type semen samples was performed to interrogate this hypothesis. The number of available semen samples of Tarantino was limited and, therefore, it was decided not to include these valuable samples. However, if ABHD16B would have an effect on lipid biosynthesis during spermatogenesis, this should also be detectable in heterozygous samples. After normalization to 10^6^ sperms per sample, no significant difference in the total lipid content between 15 wild type and 15 heterozygous samples was detected (Figure 5).

**Figure 6:**
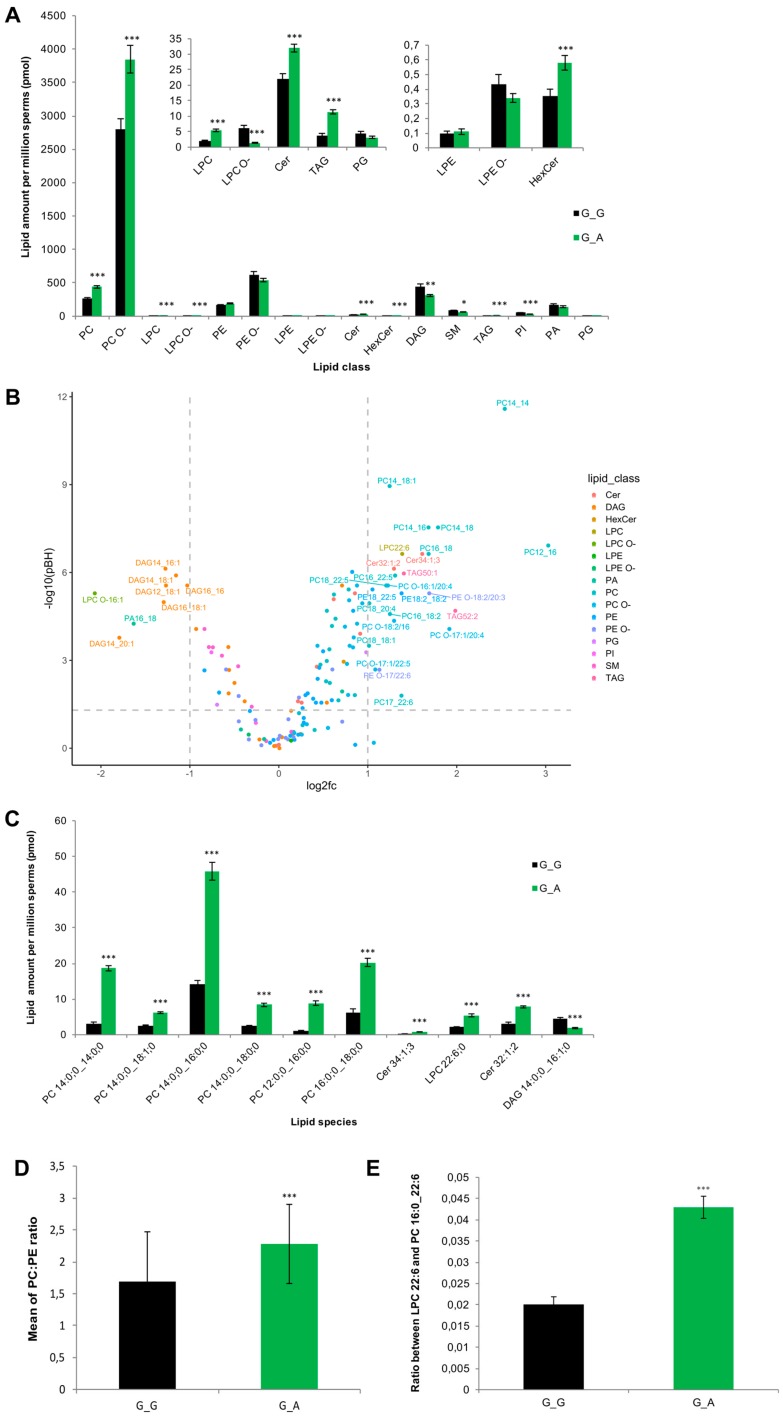
Lipid distribution and variance of spermatozoa in wild type and heterozygous sires. (**A**) Change profile of total lipid content for each lipid class of sperms in two genotypes. (**B**) −log_10_ of adjusted *p*-value (p_(BH)_) and mean log_2_-fold change (G_A vs G_G) of 144 lipid species were plotted, lipid species with −log_10_p_(BH)_ >1.3 and |log_2_fold change|>1 (dashed grey lines are included) were annotated with lipid feature names (simplified without the saturated acyl groups). (**C**) Top 10 most significantly changed lipid species between G_G and G_A bull sperm cells. (**D**) Bar chart shows the mean of PC:PE ratio in G_G and G_A bull sperms. (**E**) Bar chart shows the ratio between LPC 22:6 and PC 16:0_22:6 in two genotypes. G_G: wild type; G_A: heterozygous. Differential changes were tested by the Mann–Whitney U test or Student’s t-test. Data are presented as mean ± SEM; *n* = 15/group; FDR adjusted *p*-values are indicated: * *p* < 0.05, ** *p* < 0.01, *** *p* < 0.001.

However, 10 out of 16 lipid classes showed significant differences (Figure 6A). The majority of different lipids belonged to the classes of diacylglycerols (DAG), glycerophosphocholines (PC, PC O-), ceramides (Cer), and sphingomyelins (SM). The sperms of heterozygous carriers showed significantly decreased amounts of SM and DAG, while PC, PC O-, and Cer were increased (Figure 6A). In total, 99 of 144 lipid species demonstrated significant differences between wild type and heterozygous sperm samples. Eight lipid species significantly decreased (*p_(BH)_*<0.05, log_2_fc < −1), six of them were DAGs. 25 lipid species significantly increased (*p_(BH)_* < 0.05, log_2_fc > 1), almost half (*n*=12) of them were PCs (Figure 6B). Figure 6C shows the ten most significantly changed lipid species. An important indicator of cell membrane integrity is the PC:PE ratio. As shown in Figure 6D, heterozygous sperms have a significantly increased the PC:PE ratio. Another sperm membrane structure criterion is the ratio between LPC 22:6 and PC 16:0_22:6, and it also significantly increased in the heterozygous samples (Figure 6E).

## 3. Discussion

Male infertility is a complex multifactorial idiopathic, congenital, or acquired heterogeneous disease [37,38]. In men, genetic factors predominantly cause idiopathic conditions contributing to 30–40% of male infertility [39]. However, up to now, for only three genes, i.e., *NR5A1*, *DMRT1,* and *TEX11*, associations with male infertility have been evidenced in independent biological and functional studies [40]. The same number of genes has been identified in cattle causing bull sub- or infertility so far, i.e., *FSHB* (BTA15, 61.7 Mb), *TMEM95* (BTA19, 27.6 Mb), and *ARMC3* (BTA13, 24.3 Mb) [27,28,29,30]. Although the exact chromosomal positions of these genes differ from precise infertility associated chromosomal regions that have been identified either by QTL studies, GWAS using SCR as parameter, or whole exome sequencing, they are located on the same chromosomes [24,26,41]. Regarding the location of *ABHD16B* on BTA13, it is noteworthy that not only *ARMC3* is located on the same chromosome, but also QTLs for percentage of normal sperms (68.18 cM), male fertility (43.76 cM), and non-return rate (EBV) (85.19 cM) have been mapped to BTA13 [42,43,44]. One region explaining roughly 0.6% of the genetic variance of SCR was detected on BTA13 from position 58,456,868–59,951,247 harboring two potential candidate genes for male fertility, i.e., *CTCFL* and *SPO11* [45]. This region is located approximately 4 Mb downstream of *ABHD16B*. The closest SNP identified by whole exome sequencing was located on BTA13 at position 53,691,419 within the *SIRPA* gene only 738,396 bp upstream of the nonsense variant in *ABHD16B*. In a further GWAS using a much larger dataset (11.5 k Holstein bulls) and higher density SNP chip (about 300 k), five markers with marked dominance effects were detected, one of them being located on BTA13 (13:g.60263194A>C; rs41701032) [46]. Hence, the molecular genetic data that are published elsewhere are well in agreement with our findings.

The identification of *ABHD16B* as an associated causative gene for bull infertility also allowed for us to elucidate its biochemical function. Except that ABHD16B belongs to a large protein superfamily of catalytic enzymes harboring an α/β-hydrolase domain and is predominantly expressed in Leydig cells of the testis, nothing was known regarding its biochemical or physiological function so far [31]. The methylation of *ABHD16B* was reported to be associated with chronic obstructive pulmonary disease (COPD) and aberrant methylation patterns were identified in infertile man [32,36]. In proteome studies, 11 members of the ABHD family (1, 2, 5, 6, 10, 11, 12, 13, 14B, 16A, 17A, 17B) have been detected in testis or spermatozoa [47,48]. Human ABHD2 participates in sperm hyperactivation as a lipid hydrolase through depleting endocannabinoid 2-arachidonoylglycerol (2-AG), an inhibitor of sperm calcium channel (CatSper) [49]. However, in most cases, their exact role remains elusive.

Our data show that ABHD16B is involved in lipid biosynthesis of DAGs. According to the ABHD16B molecular structure, it is supposed to participate in lipid metabolism, like other ABHD family members, which could contribute to sperm maturation. Sperm lipidomics of two different genotypes was performed to confirm this. Sperm lipid composition changes during their maturation through the epididymis, the percentage of SM in spermatozoa increased [50,51]. SM is synthesized by the combination of Cer and phosphorylcholine from PC. During this reaction, DAG is produced as a by-product [52]. Our data demonstrated that SM and DAG were significantly decreased, while PC and Cer were significantly increased in heterozygous spermatozoa. This implies that ABHD16B might be involved in the lipid biosynthesis of DAG, which influences SM synthesis in the later process. On the other hand, increased levels of PCs in heterozygous sperms could also result from an inhibited degradation from PC to DAG and phosphorylcholine. Cer increased correspondingly without enough phosphorylcholine combined to synthesize SM.

The presence of ABHD16B in the epididymis, as shown by IHC, suggests a role in the lipid metabolism of DAG and SM during sperm maturation. DAG also influences the synthesis of 2-AG, which is an inhibitor of sperm calcium channel (CatSper) preventing sperm hyperactivation. DAG is hydrolized to 2-AG by diacylglycerol lipase (DAGL) and, hence, decreased DAG levels in homozygous carrier sperms could result in an insufficient amount of 2-AG leading to a premature capacitation [53]. Furthermore, this effect could be enhanced by the lack of SM. On the other hand, the accumulation of PC and Cer could also interfere with the fertilization capacity. For instance, increased PC concentrations in chicken sperms were reported to be negatively associated with fertility during aging [54]. It has also been observed that imbalanced lipid homeostasis of PC and SM caused sperm membrane instability and infertility in knockout mice [55]. The final sperm lipid composition is formed during epididymal maturation, which results in a decreased amount of cholesterol, PS, CL, PE, and PI, and an increase in PC and DAG. The amount of PI, PC, and DAG was significantly different between the wild type and heterozygous variant spermatozoa (Figure 6A), indicating a potential role of ABHD16B in sperm maturation.

Another impact of ABHD16B on lipid metabolism can be seen in the increased PC:PE ratio. Abnormal PC:PE ratios affect membrane permeability, fluidity, and integrity [56,57]. In cells that have abundant unsaturated fatty acids, such as spermatozoa, LPC is normally regarded as a marker of sperm membrane quality and oxidative stress. The increase of LPC content in the deteriorated membrane of spermatozoa indicates affected acrosome reaction, and an increased ratio between LPC 22:6 and PC 16:0/22:6 was observed in human spermatozoa with impaired membrane [58]. The ratio was also significantly enhanced in the heterozygous samples analysed here. Furthermore, LPC 22:6 is a reliable marker of spermatozoa lipid oxidation [59]. A significant increased concentration of LPC 22:6 was also found in heterozygous carrier samples. This could result in a higher oxidized state or membrane damaged level in contrast to wild type sperms. The lipidomics analysis clearly showed that the loss of ABHD16B function has a profound effect on sperm plasma membrane lipid composition. Therefore, in analogy with experiments in humans and mice, it can be hypothesized that the altered lipid composition of the *ABHD16B* homozygous carrier sperms interferes with the fertilization ability.

## 4. Materials and Methods

### 4.1. Ethical Statement

EDTA blood samples of cattle were taken for routine parentage control exclusively by local veterinarians. The Lower Saxony State Office for Consumer Protection and Food Safety approved the collection of samples (33.19-42502-05-17A196), according to §8a Abs. 1 Nr. 2 of the German Animal Protection Law.

### 4.2. Genome Wide Association Analysis (GWAS)

The conception ability of sires (non-return rate, NR*_dev_*) was calculated based on the latest three daughter proven service-sire age groups (2019: A.I. sires born 2014–2016; 0% deviation). The NR*_dev_* is expressed in %-deviation on the original non-return-rate scale. Sires in the breeding population with NR*_dev_* values of approx. ± 2% are scored as average (for more information see https://www.vit.de/fileadmin/DE/Zuchtwertschaetzung/Zws_Bes_eng.pdf). Data of NR*_dev_* deviations of service-sires were provided by VIT (https://www.vit.de/en/).

For GWAS 279 sires of the current breeding population were randomly chosen as presumably fertile controls. As cases 10 sires (including Tarantino, NR*_dev_* = −29) with NR*_dev_* between −29 and −2 were selected (Table 1). The 289 samples were genotyped while using the Illumina BovineSNP50 or MD BeadChip. The chips were processed on a HiScan SQ and iScan System (Illumina GmbH, Munich, Germany) and raw data were converted using GenomeStudio Software (Illumina GmbH, Munich, Germany). Final reports were imported into SVS 8.8.3 for MacOSX (Golden Helix Inc. Bozeman, MT, USA). Prior to GWAS data were filtered while using a call rate <0.95, number of alleles >2, minor allele frequency (MAF) <0.05, and Fisher´s HWE <0.001 (based on controls) as marker dropping criteria. LD pruning was performed with a window size of 100 and increments of 5. *R*^2^-LD statistics with a threshold of 0.5 while using Cochran-Mantel-Haenszel (CHM) as computation method was applied. After filtering, 38,671 markers remained for further analysis. GWAS was done using a multi-locus mixed model (MLMM) while applying an additive genetic model with correction for male X-chromosomal hemizygosity [60,61]. The associations were regarded as statistically significant above a Bonferroni threshold of −log_10_*P* = 5.9 (*p* = 0.01). The associations of markers (−log_10_*P*-value, y-axis) were plotted against their chromosomal positions (UMD3.1.1, x-axis).

### 4.3. Next Generation Sequencing of Tarantino and Its Parents

Tarantino and its parents were sequenced on a HiSeq2500 System (Illumina GmbH, Munich, Germany), resulting in approx. 10^9^ total reads per sample. Low quality (average phred quality < 15) and single reads were removed, resulting in approx. 9.4 × 10^8^ per sample. Mapping to the bovine reference genome sequence (UMD3.1.1) was done while using BWA [62]. PCR duplicates were removed using Picard (http://broadinstitute.github.io/picard/). After read mapping, alignment and refinement approx. 7.8 × 10^8^ reads remained per sample, corresponding to an average depth of coverage of approx. 46x (mean insert size 360 bp). A total of 9,315,126 SNPs and 1,439,972 indels were called using GATK Haplotype Caller [63]. SNP & Variation Suite 8.8.3 (Golden Helix Inc., Bozeman, MT, USA) was used for further analysis. SNPs and indels were set to missing with read depth ≤ 10, genotype quality ≤ 15, alt read ratios for Ref_Ref ≥ 0.15, Ref_Alt outside 0.3 and 0.7, Alt_Alt ≤ 0.85, and according to their inheritance pattern (Tarantino = Alt_Alt, parents Alt_Ref). After this filtering, 307,898 SNPs and 604 indels remained. A final filtering was done while using SNPs and indels only in annotated and verified mRNA transcripts, including splice donor and acceptor distances of 2 bp, splice region exonic distances of 3 bp and splice region intronic distances of 8 bp, resulting in 78,472 SNPs and 125 indels.

### 4.4. Genotyping of SNP rs468948776 (ABHD16B)

The nonsense variant in *ABHD16B* was genotyped while using fluorescence resonance energy transfer (FRET) analysis on a LightCycler 480 (Roche Life Science, Mannheim, Germany). The DNA concentrations were measured using NanoDrop ND-1000 spectrophotometer (PEQLAB Biotechnologie GmbH, Erlangen, Germany). Conventional PCR primers were designed using the online program Primer3 (http://bioinfo.ut.ee/primer3-0.4.0/). The FRET primers were designed with MeltCalc Software [64,65]. Table 2 lists FRET primers and probes.

SNP rs468948776 (*ABHD16B*) was amplified in a total volume of 25 µL, including 20 ng DNA, 10 µmol forward and reverse primer each, 10 µmol probe and anchor (Sigma-Aldrich, Taufkirchen, Germany) each, 1 × GC-RICH solution, 1 × PCR reaction buffer (including 20 mM MgCl_2_), 100 μmol dNTPs and FastStart Taq Polymerase (1U; Qiagen, Hilden Germany) for 34 cycles at 95 °C for 15 S, 60 °C for 20 S, and 72 °C for 20 S. The melting curves were done using the following program: 95 °C for 30 S, 37 °C for 30 S, 95 °C continuous acquisition mode (2/°C), ramp rate 0.29 °C/S, followed by 37 °C for 30 S.

### 4.5. Western Blotting

Immunoblotting on cryopreserved semen specimens of one wild type (G_G), one heterozygous carrier (G_A), two homozygous affected (A_A; Tarantino, C_a_), and testis, muscle, and liver samples of wild type bulls were prepared. The semen samples of sire C_a_ were provided from the safety-inventory of a AI station. Human ABHD16B over-expression lysate (NM_080622, OriGene, Rockville, MD, USA) was used as a positive control. Frozen semen samples were thawed at 37 °C in a water bath for 30 S., followed by 3 × washes with phosphate-buffered saline (PBS; Invitrogen/ThermoFisher Scientific) and lysed in cold RIPA buffer (Sigma, R0278, St. Louis, MO, USA). Protease inhibitor (Roche, Cat. No.04693159001, Mannheim, Germany) and phosphatase inhibitor (Roche, Cat. No. 04906845001, Germany) were added to RIPA buffer in advance. The samples were incubated for 1 h at 4 °C and centrifuged at 16,000× *g* for 20 min. at 4 °C. An additional homogenization of tissue samples with MagNA lyser green beads (Roche Life Science, Mannheim, Product No. 03358941001, Germany) was carried out followed by an incubation for 2 h at 4 °C and then centrifuged at 16,000× *g* for 20 min. at 4 °C. Protein quantification was performed by Bradford method with the dye reagent concentrate (Bio-Rad, Cat. No. 5000006, Munich, Germany).

After denaturation (10 min. at 70 °C) in LDS sample buffer with 5% 2-mercaptoethanol, equal amounts of protein were loaded to SDS-PAGE (8% Bis-Tris Plus gel, ThermoFisher Scientific, Cat. No. NW00087BOX, USA). After electrophoresis at 15V for 1h, the proteins were transferred onto nitrocellulose membranes (Sigma, Cat. No. 10600098, Germany) with semi-dry blotter (Brenzel Bioanalytik, Lahntal, Germany). Membranes were blocked with 5% non-fat dry milk in TBS-T (0.1% Tween) overnight at 4 °C and then incubated with primary antibodies for 1 h at room temperature, followed by incubation with the secondary antibodies at room temperature for 1 h. Subsequently, the membranes were incubated with an ECL detection reagent (GE Healthcare, Product No. RPN2109, Little Chalfont, UK) and then exposed to X-ray films (GE Healthcare, Product No. 28906836, Tokyo, Japan) for detection.

A customized bovine ABHD16B primary antibody, affinity purification PAC-DFR (Davids Biotechnologie GmbH, Regensburg, Germany, 1 µg/mL dilution) was used. Goat Anti-Mouse IgG (H + L)-HRP (Bio-Rad, Munich, Germany; 1:10,000 dilution) and Goat Anti-Rabbit IgG (H + L)-HRP (Bio-Rad, Germany; 1:10,000 dilution) were the secondary antibodies. Anti-α-Tubulin (Sigma, T9026; 1: 2500 dilution) was used as the loading control.

The quantification of ABHD16B Western blots was done using ImageJ 1.52k software [66]. Areas under curve of ABHD16B specific bands were determined for liver, muscle, testis and spermatozoa (G_G). Relative expression ratios (%) were calculated with α-tubulin as the internal standard and plotted as Box and Whisker plot.

### 4.6. Immunohistochemistry of Testes

Testicular and epididymal tissues were obtained from freshly slaughtered wild type Holstein cattle and they were immediately fixed in 4 % formaldehyde for 48 h. Immunohistochemistry (IHC) was performed on paraffin-embedded sections, including testicular parenchyma, as well as ductuli efferentes, epididymal head, corpus, and tail with efferent ducts and epididymal duct, respectively. The primary polyclonal antibody was directed against the PAC-ARK peptide and it was generated in the rabbit according to standard protocols (Davids Biotechnologie GmbH, Regensburg, Germany). IHC was performed in an automated immunostaining system (Discovery XT, Roche Diagnostics GmbH, Mannheim, Germany) at a dilution of 1:1000 while using the SABC (streptavidin-biotin-complex) method, mild EDTA (ethylenediaminetetraacetic acid) pretreatment, and DAB (diaminobenzidine tetrahydrochloride) for signal detection (DAB Map Kit, Roche Diagnostics GmbH, Mannheim, Germany). A rabbit IgG isotype control (ABIN3023746, antibodies-online GmbH, Aachen, Germany) was included at the same concentration as the primary antibody for confirmation of primary antibody specificity. Additionally, pure antibody diluent instead of primary antibody was applied to the control sections for an evaluation of non-specific binding of the secondary antibody.

### 4.7. Lipidomics of Wild Type and Heterozygous Spermatozoa

#### 4.7.1. Semen Collection for Lipidome Analysis

Wild type and heterozygous fresh semen samples were prepared for lipidome analysis. Three independent fresh ejaculates were collected from a heterozygous bull and five technical replicates were produced by dilution from each sample. Wild type semen samples were flushed from the epididymal tail of four unrelated bulls and a total of 15 technical replicates were generated by dilution. In the epididymal tail, spermatozoa are matured and the lipid composition is equivalent to ejaculated spermatozoa [67,68]. The samples were washed twice in Dulbecco’s phosphate-buffered saline (D-PBS) without magnesium and calcium and centrifugated at 1000× *g* for 5 min. at 4 °C. The cells were resuspended in D-PBS to a final concentration of approximately three million–eight million cells/mL. Cell density was determined in an improved Neubauer counting chamber (Marienfeld GmbH, Lauda-Königshofen, Germany).

#### 4.7.2. Lipid Extraction for Mass Spectrometry Lipidomics

Mass spectrometry-based lipid analysis was performed by Lipotype GmbH (Dresden, Germany), as described [69]. The lipids were extracted while using a two-step chloroform/methanol procedure [70]. The samples were spiked with internal lipid standard mixture containing: cardiolipin 16:1/15:0/15:0/15:0 (CL), ceramide 18:1;2/17:0 (Cer), diacylglycerol 17:0/17:0 (DAG), hexosylceramide 18:1;2/12:0 (HexCer), lyso-phosphatidate 17:0 (LPA), lyso-phosphatidylcholine 12:0 (LPC), lyso-phosphatidylethanolamine 17:1 (LPE), lyso-phosphatidylglycerol 17:1 (LPG), lyso-phosphatidylinositol 17:1 (LPI), lyso-phosphatidylserine 17:1 (LPS), phosphatidate 17:0/17:0 (PA), phosphatidylcholine 17:0/17:0 (PC), phosphatidylethanolamine 17:0/17:0 (PE), phosphatidylglycerol 17:0/17:0 (PG), phosphatidylinositol 16:0/16:0 (PI), phosphatidylserine 17:0/17:0 (PS), cholesterol ester 20:0 (CE), sphingomyelin 18:1;2/12:0;0 (SM), and triacylglycerol 17:0/17:0/17:0 (TAG). After extraction, the organic phase was transferred to an infusion plate and dried in a speed vacuum concentrator. First step dry extract was re-suspended in 7.5 mM ammonium acetate in chloroform/methanol/propanol (1:2:4, *V*:*V*:*V*) and second step dry extract in 33% ethanol solution of methylamine in chloroform/methanol (0.003:5:1; *V*:*V*:*V*). All liquid handling steps were performed while using Hamilton Robotics STARlet robotic platform with the Anti Droplet Control feature for organic solvents pipetting.

#### 4.7.3. MS Data Acquisition

The samples were analyzed by direct infusion on a QExactive mass spectrometer (Thermo Scientific, Osterode am Harz, Germany) equipped with a TriVersa NanoMate ion source (Advion Biosciences, Ithaca, NY, USA). Samples were analyzed in both positive and negative ion modes with a resolution of Rm/z=200=280000 for MS and Rm/z=200=17500 for MSMS experiments, in a single acquisition. MSMS was triggered by an inclusion list that encompasses corresponding MS mass ranges scanned in 1 Da increments [71]. MS and MSMS data were both combined to monitor CE, DAG, and TAG ions as ammonium adducts; PC, PC O-, as acetate adducts; and, CL, PA, PE, PE O-, PG, PI, and PS as deprotonated anions. MS only was used to monitor LPA, LPE, LPE O-, LPI, and LPS as deprotonated anions; Cer, HexCer, SM, LPC, and LPC O- as acetate adducts.

#### 4.7.4. Data Analysis and Post-Processing

The data were analyzed with in-house developed lipid identification software based on LipidXplorer [72,73]. Data post-processing and normalization were performed while using an in-house developed data management system. Only lipid identifications with a signal-to-noise ratio >5, and a signal intensity five-fold higher than in corresponding blank samples were considered for further data analysis.

The total lipid amount occurring in each sperm sample were pre-tested to ensure that optimal amounts are used to achieve the greatest analysis quality and result comparability, despite the broad dynamic range of our analytical methods. Afterwards, the initially detected total lipid amount per sample was normalized to one-million sperms. A significant difference between normalized total lipid amount of wild type and heterozygous samples was analyzed.

A 70% occupational threshold was applied for data filter, valid data in more than 10 samples for each genotype were selected, NAs were replaced with zeros. Afterwards, lipid data that were present in both genotypes were chosen for further analysis. In total, 16 lipid classes with 144 lipid species were evaluated, and the data were analyzed in terms of lipid class and species separately. Shapiro–Wilk Test was used for normal distribution detection [74]. Significant difference analysis was performed with Mann–Whitney U-test or two-tailed t-test, depending on the normal distribution results by SPSS 16.0. PC:PE and (LPC 22:6):(PC 16:0_22:6) ratios were also checked for significant difference. Benjamini & Hochberg method was used for *p*-value adjustment of multiple testing [75] with R version 3.5.1, *p_(BH)_* < 0.05 were considered to be statistically significant (Table S1). The comparison results of lipid classes and 10 most significantly changed lipid species between wild-type and rs468948776 heterozygous samples were demonstrated in histograms (data are presented with mean ± standard error of mean). 144 lipid species were plotted with Y-axis of adjusted values (−log_10_pBH) against X-axis of log_2_fold change (heterozygous vs wild type).

## 5. Conclusions

We have identified a nonsense mutation in the bovine *ABHD16B* gene as a potential causative protein-altering variant for male infertility in Holstein cattle. This made it possible to elucidate the so far unknown physiological and biochemical role of ABHD16B in lipid biosynthesis, spermatogenesis, and fertilization. Our findings could also have implications on further elucidating a novel genetic cause for human male infertility, due to the fact that a number of deleterious variants, e.g., missense, frameshift, indels and one stop-gain variant in the human *ABHD16B* gene have been reported to the human ENSEMBL database.

## Figures and Tables

**Table 1:** Sub- and infertile sires selected for genome-wide association analysis.

Sire/ID	NR*_dev_*^a)^	No. of First Inseminations
Tarantino	−29	412
19_39644	−27	402
19_39643	−25	364
05_34345	−9	412
04_44565	−4	421
04_39067	−3	315
04_43327	−3	424
04_40476	−2	407
04_41962	−2	640
04_37666	−2	571

a) NR*_dev_*: Non-return rate deviation.

**Table 2:** Fluorescence resonance energy transfer (FRET) primers and probes used for genotyping of *ABHD16B* variant.

Gene	Primer Name	Sequence (5’->3’)	Probe Name	Sequence (5’->3’)
*ABHD16B*	ABHD16B_FRET_f	ACCCGGGCTTCGGGGGCAGC	ABHD16B_FRET_Pro	[Cy5]CGTTCCCTCAGCATGATG[Phos]
	ABHD16B_FRET_r	GCGTACTTGACCACCACGTC	ABHD16B_FRET_Anc	GGGGCAGCACGGGCG[Flc]

## Supplementary Materials

Supplementary materials can be found at https://www.mdpi.com/1422-0067/21/2/627/s1.
